# Online Relationships and Social Media Interaction in Youth Problem Gambling: A Four-Country Study

**DOI:** 10.3390/ijerph17218133

**Published:** 2020-11-03

**Authors:** Iina Savolainen, Markus Kaakinen, Anu Sirola, Aki Koivula, Heli Hagfors, Izabela Zych, Hye-Jin Paek, Atte Oksanen

**Affiliations:** 1 Faculty of Social Sciences, Tampere University, 33014 Tampere, Finland; anu.sirola@tuni.fi (A.S.); heli.hagfors@tuni.fi (H.H.); atte.oksanen@tuni.fi (A.O.); 2 Institute of Criminology and Legal Policy, University of Helsinki, 00014 Helsinki, Finland; markus.kaakinen@helsinki.fi; 3 Department of Social Research, University of Turku, 20500 Turku, Finland; akjeko@utu.fi; 4 Department of Psychology, University of Córdoba, 14004 Córdoba, Spain; izych@uco.es; 5 Department of Advertising & Public Relations, Hanyang University, Ansan 15588, Korea; hjpaek@gmail.com

**Keywords:** problem gambling, online relationships, social media interaction, youth

## Abstract

The objective of this study was to examine if belonging to online communities and social media identity bubbles predict youth problem gambling. An online survey was administered to 15–25-year-old participants in the United States (*N* = 1212), South Korea (*N* = 1192), Spain (*N* = 1212), and Finland (*N* = 1200). The survey measured two dimensions of online behavior: perceived sense of belonging to an online community and involvement in social media identity bubbles. Belonging to an online community was examined with a single item and involvement in social media identity bubbles was measured with the six-item Identity Bubble Reinforcement Scale. The South Oaks Gambling Screen was used to assess problem gambling. Statistical analyses utilized linear regression modeling. According to the analyses, strong sense of belonging to an online community was associated with higher problem gambling, but the association was observed mainly among those young individuals who were also involved in social media identity bubbles. For those youths who did not indicate identity bubble involvement, online relationships appeared to function as those offline. Some differences across the four countries were observed but overall, the results indicate that social media identity bubbles could partly explain the harmful influence that some online relations have on youth behavior.

## 1. Introduction

The Internet and social media have remarkably shaped the world and people’s everyday lives in a relatively short amount of time. Communication and information flow which used to require a considerable amount of time and were tied to physical locations can now happen in an instant and without the limitations of place, distance, or time zones. This societal and technological change impacts people of all ages, but its effects can be observed particularly among the younger generations often referred to as the digital generation or digital natives [1,2], as they have grown up with the Internet and advanced technology. These technological developments have also impacted the gambling industry, as the Internet has allowed gambling businesses to expand their services through different online platforms on a multitude of devices [3,4]. Consequently, gambling opportunities have increased considerably and many youths of today engage in gambling activities [5,6,7]. Given that the technological developments and the growth of the gambling industry are concurrent, this cross-national study set to investigate if adolescents’ and young adults’ online relationships and social media interaction predict youth problem gambling.

Adolescence and young adulthood are typically considered as times of transition and increased independence [8,9,10]. During these years, young individuals begin to explore social interactions outside the family and construct meaningful relationships with peers [11,12]. As today’s adolescents and young adults spend significantly large amounts of time online, the structure of their social life changes. This manifests in lessened traditional group interactions and increased interactions accomplished through virtual communication [13,14,15]. Young people apply advanced technology adeptly and are skilled Internet users, making it easy to find and connect with like-minded others on social media and online space at large [16,17].

People are innately social and seek companionship and social interaction when and where achievable [18,19]. For the digital generation, online communities offer new ways of taking part in peer activities and become members of groups. Online communities can represent important places for company, friendship, information, and support [20,21]. Constructing one’s individual identity and social identity through online groups or communities is another development offered by the Internet and modern technology. While online groups and communities can have many benefits for a young individual, such as providing social capital, developing and maintaining social relationships, and enhancing well-being [20,22,23], the outcomes of online networking are not all positive. For instance, social media use has been associated with depression, lower self-esteem, decreased academic achievement, and poor sleep quality (e.g., [24,25,26]).

Active use of social media may also lead to the formation of identity-driven online cliques called social media identity bubbles [27]. The concept of social media identity bubbles is based on the identity bubble reinforcement model (IBRM) originally introduced by Keipi and colleagues [27]. Social media identity bubbles are a result of social media interaction and based on shared identity, social homophily, and reliance on information from one’s online networks [28]. These networks are limited in diversity of social contacts and information received due to selectivity and filtering technologies of different social media platforms [29,30,31]. This type of one-sided interaction is narrow and can impact one’s attitudes, beliefs, and behavior [27,28,32]. However, little is known so far how involvement in these identity-driven social media identity bubbles might influence youth behavior, especially in terms of risk behavior such as gambling, which has become an increasingly popular activity among young individuals worldwide [6,33,34].

Adolescents and young adults are generally known to take risks and do things that can harm their health [35,36]. The odds of voluntarily acquiring unhealthy habits such as substance use or taking part in behaviors that involve a high likelihood of personal injury (e.g., fighting or speeding) increase throughout the course of development but are particularly pronounced during the teenage years [36,37]. Furthermore, DiClemente et al. [38] predicted in 2013 that in the future adolescents’ risk behaviors would become progressively problematic. Indeed, new forms of information technology and social media have brought along additional challenges in terms of youth behavior and risk-taking. Previous research has found that increased use of technology and screen-time can lead to compulsive Internet use [39], higher exposure to cyberbullying [40], and exposure to content that can be unsuitable and distressing, or promote risk-taking (i.e., content that is sexual or violent in nature, focuses on potentially illegal activities, or advertises alcohol or tobacco products [41]).

Research further suggests that engaging in different risk behaviors is more likely among those youths with close ties to peers online [42,43,44], whereas strong relationships offline seem to be linked to decreased risk behavior [45,46]. It is possible that social relationships online are fundamentally and qualitatively different from those constructed offline, and thus influence youths’ behavior and beliefs in unanticipated ways. Easy access to like-minded others who empathize with one’s opinions might become an important extension of one’s identity, but lack essential features like social support, authentic feedback, and exposure to contrasting views that are more inherent to offline relationships [47,48]. Consequently, strong sense of belonging to online communities and subsequent involvement in social media identity bubbles may at least partially explain why online social networks function differently from those offline. However, more research is needed to recognize what makes the differences between online and offline social relationships.

### 1.1. Social Media Identity Bubbles and the Identity Bubble Reinforcement Model

Online networks represent a plentiful and efficient structure of interaction where people can create and distribute content at any time, as well as choose the type and intensity of their interaction [45,46]. Social media sites and platforms such as Facebook, Instagram, and Twitter exemplify online networking channels where users can effortlessly connect with similar-minded others and generate and distribute content at desired frequency [47,48]. While online content is immeasurable in nature, due to sophisticated filtering mechanisms, media content at large and the content seen on individuals’ social media sites can become highly personalized [49]. This type of personalization of content leads to selective exposure which, in turn, can influence people’s opinions and have adverse effects on, for instance, political discourse and open-mindedness [30,49].

The phenomenon of personalization of online content has been examined and discussed under such terms as echo chambers and filter bubbles [30,50]. Echo chambers refer to self-selected personalization; a situation where among a diverse range of information, an individual chooses and consumes content that expresses and supports their viewpoint [49,50,51]. Filter bubbles, on the other hand, occur through pre-selected personalization where the individual is not deliberately choosing content, but based on the individual’s browsing activity, the content is filtered by websites or advertisers through intricate algorithms [30]. Echo chambers and filter bubbles alike decrease the likelihood of encountering ideologically diverse and cross-cutting information, leading to polarization and biases in both information and confirmation [52,53,54,55,56,57,58].

In an effort to research and understand the rapidly changing media landscape, concepts like echo chambers and filter bubbles are important. However, as such, echo chambers and filter bubbles are grounded on computer science and measure personalization of content and online bubbles merely structurally. Consequently, they do not account for more socio-cognitive aspects of the phenomenon. The identity bubble reinforcement model (IBRM) challenges this shortcoming and attempts to fill the existing gap in social media selectivity literature by integrating a social psychological perspective into the discussion [27]. The IBRM is further supported by Kaakinen and colleagues [28] who developed and validated the identity bubble reinforcement scale (IBRS) designed to measure a person’s level of involvement in social media identity bubbles. According to the IBRM, online behavior and communication are identity-driven processes, because individuals search for interaction with others who share similar features with them and thus validate their identities [27].

Social media identity bubbles, as proposed by the IBRM, are a psychosocial concept of online interaction and they are constructed of three interrelated elements: identification with social networks, predisposition to interact with similar others, and relying on information provided by one’s social networks [27,28]. Even though social media provide endless possibilities for networking and communication, people have the capacity to control and choose whom they interact with and can easily manage the connections they built. This enables the formation of exclusive online cliques, i.e., social media identity bubbles, that are formed around similar individuals, interests, values, activities, or ideologies [32]. While literature based on computer science often examines online group phenomena through specific online platforms and discussions, the IBRM helps explain how individuals relate themselves with groups and information online [28].

Interacting with like-minded individuals provides grounds for social identification processes but also tends to lead to increased homophily. This, in turn, leads to increased trust in information provided by one’s constructed social group. These processes support the mutually reinforcing nature of social media identity bubbles. Social media identity bubbles are based on basic human motivation for identity validation but they can also create new kinds of problems. Exposure to one-sided interaction with others who share similar attitudes, identities, and biased information can reinforce harmful and risky behaviors among online community members [27]. Given that research on social media identity bubbles is still in its early stages, more investigation on their impact on individuals is needed. This social psychological study uses the IBRM as a framework and investigates the role of social media identity bubbles in youth problem gambling.

### 1.2. Youth Problem Gambling

Gambling involvement at large and gambling as a recreational activity have gained popularity among young individuals over the past decade [6,59,60]. Even though legislative statutes globally prohibit gambling activities from underage individuals (those under the ages of 18 or 21 in most countries [5,61,62]), Internet gambling and sophisticated technologies have expanded gambling opportunities significantly. Due to this dramatic change of the gambling industry, gambling has become more visible to young people and made different forms of gambling accessible to young—even underage individuals by bypassing the statutory age restriction efforts [3,63]. Young people are the most active Internet users and they spend a significant amount of time online [52,64]. Consequently, they can become exposed to risky content, like that involving gambling, through various settings, including online video games and social networking services, or actively seek for such content or communities from numerous sites [4,65,66,67]. Those youths who have strong and supportive offline relationships might be more resilient and better prepared to face potentially harmful online content, while youths with strong online relationships might be more vulnerable to various risk behavior content and more susceptible to participating in risk behavior [44,46].

In the wake of the new gambling environment, research has also recognized the existence of different online communities that are formed around gambling themes and discussions [66,68,69]. The interactions and shared activities taking place in these online communities may bias normative behavior and further encourage youth gambling practices. This may be evident in the recent youth problem gambling statistics. Indeed, recent body of research indicates that youth begin to gamble before they start experimenting with substances such as tobacco and alcohol [60]. A systematic review by Calado et al. (2016) investigating the prevalence of adolescent problem gambling concluded that, worldwide, 0.2% to 12% of 11 to 24-year-olds qualify as problem gamblers and, importantly, are more susceptible to gambling online [33].

The increased popularity of gambling may have also influenced youth’s opinions about gambling as many perceive it as a socially acceptable and exciting form of entertainment [70,71]. On top of having entertainment value, the possibility to acquire wealth without much effort or extraordinary skill appeals to gamblers across age groups [72]. However, gambling can become excessive and cause problems, exceeding the perceived benefits of the activity. Gambling is typically considered to be problematic when it brings harm to the individual. Problem gambling, as discussed in this study, involves a continuum of severity and has several negative impacts on the individual, including impaired control, economic problems, emotional distress, and conflicts in social relationships [73,74]. Problem gambling is also associated with substance use [75], depression [76], and other internalizing disorders [77]. With its wide-ranging negative consequences and harms to the individual and the society alike, problem gambling has become a crucial public health issue. More research is needed to understand the possible social mechanisms behind problem gambling behavior among vulnerable young populations, while accounting for the modern virtual context.

### 1.3. The Cross-National Study

In order to investigate the significance of online belonging and social media identity bubbles in youth problem gambling cross-nationally, four countries were chosen for research comparison. These are the United States, South Korea, Spain, and Finland. These countries are culturally and geographically distinct, representing both Western and Eastern nations, as well as Nordic and southern European societies. Including countries with such fundamental variety allows us to meaningfully compare these current phenomena among youths who have grown up and live in diverse cultural settings and societies. Importantly, these four countries are similar in their technological advancements and have corresponding Internet penetration and social media usage rates, making research comparisons possible. However, some variations exist in how these countries report prevalence statistics among age-groups. For instance, in the United States, 93% of adolescents and young adults have access to the Internet. Ninety percent of young Americans between 18 and 29 years of age indicate using some form of social media [78], while approximately 63% of 15–25-year-old individuals report using the photo-sharing platform Instagram [79]. With 95% having access to smartphones, social media usage among U.S. teens is near-constant [80].

South Korea is among the leading nations when it comes to Internet penetration rate and online connectivity. Nearly all (99%) Korean young people in the 10 to 19 and 20 to 29 age groups are connected to the Internet and approximately 85% use social media [81,82]. Spain has registered steady increases in Internet penetration over the last decade. In the year 2017, 83% of Spanish households reported having Internet access [83]. Spanish youths are active social media users with 90% of 16–24-year-olds actively participating in social media networks [84]. Correspondingly in Finland, all (100%) 16–24-year-old young individuals have access to the Internet and nearly 93% of individuals in this age group use social media platforms [85].

These countries also share similarities in gambling prevalence profiles and recognize the challenge of youth problem gambling, even though engaging in various gambling activities is illegal for underage individuals in all of them. In the United States, the legal gambling age varies between different states, but is typically between 18 and 21 years of age [62]. However, 6%–15% of youth in the country experience problems related to gambling and 2%–7% have a gambling addiction [86]. In South Korea, the legal gambling age is 18 years [87]. It is estimated that up to 30% of individuals between 19 and 29-years of age are problem gamblers, and the corresponding rate for individuals under 19 years-old is 2.5% [88,89]. Spain has a comparatively high gambling prevalence rate with more than 76% of the general population having gambled at some point during their life, and approximately 90% of them having gambled during the past year [90,91]. Gambling is prohibited from individuals under 18 years of age in Spain [61], but according to a recent report, Spanish adolescents and young adults have the highest youth problem gambling rate in Europe [92].

In Finland, the legal gambling age is 18 years [93], and gambling prevalence is relatively high among Finnish young adults between 18 and 28 years of age. According to a Nordic comparison study, Finnish youths have a higher gambling rate than their counterparts in Denmark, Iceland, Norway, and Sweden [94]. Nearly 14% of Finnish males and 5% of females between 15 and 28 years old qualify as at-risk problem gamblers [95]. Together, these similarities and slight differences among Internet and social media usage rates, as well as gambling and problem gambling prevalence statistics make the United States, South Korea, Spain, and Finland attractive for research comparison.

Young people increasingly establish relationships online and these relationships might be distinctive from offline relationships [42,43,44,45,46]. Studies also indicate that young individuals who strongly identify with online communities show more risk behaviors [96,97,98]. Thus, we set the following hypothesis:

**Hypothesis 1 (H1).** *Strong belonging to an online community is associated with higher problem gambling among youth.*

Further, one-sided online interaction can lead young Internet and social media users to become involved in identity-driven online cliques called social media identity bubbles. Involvement in online communities combined with online identity bubbles (i.e., limited diversity of social contacts and information received) might be a risk factor for youth problem gambling. Together, they make social media influential, but one-sided social environment lacks authentic feedback and exposure to contrasting views which are important when making well-informed decisions. These protective features are more inherent to offline relationships [27,99]. Involvement in socially biased online environments may make young people more prone to risk behaviors. Thus, we hypothesize that

**Hypothesis 2 (H2).** *Involvement in social media identity bubbles moderates the relationship between belonging to an online community and problem gambling.*

**Hypothesis 3 (H3).** *The associations are constant across the four culturally distinct countries with analogous Internet penetration and social media usage rates.*

## 2. Materials and Methods

### 2.1. Participants and Procedure

The participants were 15–25-year-old adolescents and young adults from the United States (*N* = 1212, 50.17% female, M_age_ = 20.05, SD_age_ = 3.19, South Korea (*N* = 1192, 50.42% female, M_age_ = 20.61, SD_age_ = 3.24), Spain (*N* = 1212, 48.76% female, M_age_ = 20.07, SD_age_ = 3.16), and Finland (*N* = 1200, 50% female, M_age_ = 21.29, SD_age_ = 2.85). They were invited to take a 15-min online survey exploring social media use and gambling engagement. The participants were recruited from an online-panel administered by a data-provider company, Dynata (formerly known as Survey Sampling International), operating in Plano, Texas. The participants received an email with a link to our online survey.

The survey was targeted to young individuals between 15 and 25 years of age in each country, and the data were demographically balanced for age, gender, and living area, in order to make the sample characteristics match the current population estimates of individuals in this age group in each country [73]. There were no other inclusion criteria for participation in the study. In other words, the participants were not chosen based on their engagement in problem gambling or prior social media activity. This allowed us to examine the associations between youths’ online relationships and problem gambling among the general populations of the four countries and understand how impactful these phenomena are among young individuals in non-diagnostic samples. These data have been utilized in a prior study examining how social media use is related to youth risk behavior in terms of hazardous alcohol use [100].

The surveys were given in the respective language of each country. The original survey was in Finnish and it was translated to English by professional-level translators. The English survey was then translated to Korean, also by professional-level translators. Lastly, the English survey was translated to Spanish by professional-level translators. All translated surveys went through a back-translation process to confirm that the items matched accurately. Participation in the study was voluntary and all answers were anonymous. The Ethics Committee of Tampere region reviewed the research protocol before implementation and stated that the research did not involve any ethical issues. The Ethics Committee of Tampere Region: Decision 62/2016. The participants were informed they could withdraw from the study at any time. All ethical guidelines were followed.

### 2.2. Measures

Problem gambling: The South Oaks Gambling Screen (SOGS) was used to assess the level and intensity of problematic gambling behavior. The SOGS is a commonly employed screen when evaluating pathological gambling behavior [101,102]. The screen includes 20 items targeting gambling intention and engagement. The scores range from 0 to 20 with higher scores indicating potential pathological gambling. To accommodate for cultural variations in gambling in the four countries examined, we slightly modified some of the screening items. The scale had good internal consistency in all country samples with Cronbach’s alphas of 0.90 in the United States, 0.82 in South Korea, 0.79 in Spain, and 0.89 in Finland. The item was treated as a continuous variable in the analysis.

Involvement in social media identity bubbles: To examine the participants’ level of involvement in social media identity bubbles, the identity bubble reinforcement scale (IBRS-6; [28]) was employed. The scale consists of six items and three subscales: social identification, homophily, and information bias. The items (e.g., “In social media, I prefer interacting with people who are like me”) measure social media use and online group behavior. Responses were provided on a scale ranging from 1 (does not describe me at all) to 10 (describes me completely). The responses to this scale were turned into a sum variable with a range from 6 to 60. Higher score indicates higher involvement in social media identity bubbles. The scale had good to excellent internal consistency with Cronbach’s alphas of 0.90 in the U.S., 0.93 in the South Korean, 0.80 in the Spanish, and 0.80 in the Finnish sample.

Online belonging: The subjective sense of belonging to an online community was evaluated with the question, “How strongly do you feel you belong to an online community?” Answers were provided on a scale ranging from 1 (no belonging at all) to 10 (very strong belonging). We measured the participants’ sense of belonging to an online community rather than belonging to a gambling-related online community specifically. This was done in order to examine whether online relationships and belonging to an online community in general are related to problem gambling.

Offline belonging: The subjective sense of belonging to groups offline was evaluated with three questions, “How strongly do you feel you belong to: 1) a friendship group 2) family 3) school or work community?” Answers were provided on a scale ranging from 1 (no belonging at all) to 10 (very strong belonging). This variable was turned into an aggregate sum and included in the analysis as a control. Sociodemographic controls included age and gender.

### 2.3. Statistical Techniques

All analyses were performed with Stata 16 statistical software by StataCorp, College Station, Texas. First, we calculated descriptive statistics for all target variables and observed the correlations between the main variables (see Table 1 and Table 2). As a multivariate method, we utilized linear regression modeling. Two linear models were run. The first model estimated the direct effects between our target variables and included problem gambling as the dependent variable and involvement in social media identity bubbles, social belonging to an online community, and social belonging to offline groups as independent variables. To examine whether involvement in social media identity bubbles moderates the association between social belonging to an online community and problem gambling, an interaction term between social media identity bubble involvement and online belonging was included in the second model.

In order to portray the results of the interaction analysis in a more easily interpretable manner, we formed a figure in which the “Involvement in social media identity bubbles” sum variable was recoded into three categories according to the standard deviation of the aggregate data. The lowest category (labelled as “Low”) included those respondents whose sum value was not more than a standard deviation from the average. The highest category (labelled as “High”), respectively, included those respondents whose value was not less than a standard deviation from the average. Accordingly, the middle category (labelled as “Medium”) was defined for those respondents whose value ranged within a standard deviation from the average.

The regression analyses were performed separately for each individual country (the United States, South Korea, Spain, and Finland). This allowed for more stringent comparisons of the observed effects between the countries. For each model, we report the unstandardized regression coefficients (*b*), standard errors (SE), and *p*-values at the 0.05 significance level. For illustration of the interaction effects, we used the user-written coefplot package [103].

## 3. Results

According to our descriptive statistics, problem gambling was not highly common in our samples with highest scores observed among Spanish (M = 1.80, SD = 2.91) and Finnish (M = 1.60, SD = 2.56) youths, followed by U.S. youths (M = 1.27, SD = 2.55). Lowest problem gambling scores were observed among youths in the South Korean sample (M = 0.73, SD = 1.92). Using the suggested cut-off score of ≥ 5 for problem gambling measured with the SOGS [101,104], 9.32% (*n* = 113) of the U.S.; 4.03% (*n* = 48) of South Korean; 14.36% (*n* = 174) of Spanish; and 10.83% (*n* = 130) of Finnish participants were qualified as probable problem gamblers. Involvement in social media identity bubbles was highest among young individuals in the United States (M = 37.25, SD = 13.23) followed by young individuals in Spain (M = 35.82, SD = 11.85). The mean scores for involvement in social media identity bubbles were slightly lower among South Korean (M = 31.78, SD = 11.84) and Finnish (M = 27.79, SD = 9.97) young individuals.

Our direct effects linear models showed that belonging to an online community significantly predicted higher problem gambling in the United States, South Korea, and Spain. This effect was not observed in Finland. Similarly, involvement in social media identity bubbles was directly associated with higher problem gambling in the United States, South Korea, and Spain. This association was not found in Finland. Strong belonging to groups offline was significantly associated with lower problem gambling in all four countries.

**Figure 1:**
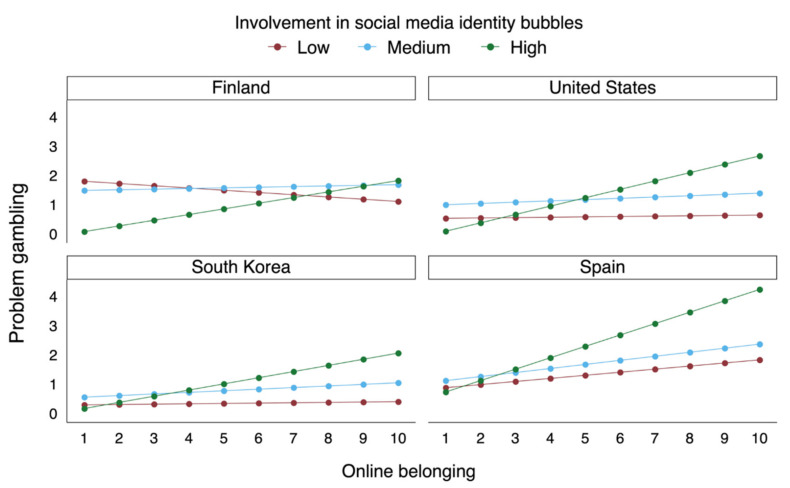
Adjusted predictions depicting the interaction between belonging to online communities and involvement in social media identity bubbles.

In terms of our interaction analyses, we discovered that strong belonging to an online community was associated with higher problem gambling behavior in the United States, South Korea, and Spain, but the association was observed mainly among those participants who were also involved in social media identity bubbles. Adjusted predictions (Figure 1) demonstrate how social media identity bubble involvement moderates the relationship between online community belonging and problem gambling. In Finland, this interaction was nonsignificant. Belonging to an online community without the impact of concurrent involvement in social media identity bubbles was not associated with problem gambling in Model 2.

Consistently across the country samples, strong belonging to groups offline was associated with lower problem gambling. Older age was associated with higher problem gambling in the United States and Spain, while male gender was a predictor of higher problem gambling in all four countries. All models are reported in full in Table 3.

## 4. Discussion

This cross-national research focused on examining young individuals’ online relationships and their role in youth problem gambling. We hypothesized that strong belonging to an online community is associated with higher problem gambling among youth, and that involvement in social media identity bubbles moderates this relationship.

According to our findings, belonging to an online community was directly associated with higher problem gambling in the United States, South Korea, and Spain, thus mainly confirming our first hypothesis. Belonging to an online community had the strongest direct effect on problem gambling among Spanish youths. This could reflect the current prevalence statistics indicating that the most active social media users in Spain are young people between 16 and 24 years of age, as well as the current state of gambling in Spain, according to which Spanish youths have the highest gambling rate in Europe [92]. It is possible that online relationships play a role in disseminating gambling behavior among Spanish young people. Likewise, involvement in social media identity bubbles had a direct positive relationship with problem gambling in the United States, South Korea, and Spain. These effects were equitable in all three countries. In Finland, belonging to an online community was not related to problem gambling. The direct effect of involvement in social media identity bubbles was also not observed in Finland. However, it should be noted that the effect of belonging to an online community became emphasized in the Finnish sample as involvement in social media identity bubbles increased. Generally, it seems that Finnish youth differ from their counterparts in the United States, South Korea, and Spain when it comes to online involvement and its relation to problem gambling. It is possible that belonging to an online community and online relationships have less weighed power in Finland and that Finnish youths place higher importance on their offline relationships. However, the overall results represent a consistent trend between online involvement and problem gambling among youth across diverse countries. This finding suggests that social media identity bubbles can be particularly influential in online interaction and youth risk behavior.

Additionally, these findings suggest that similar mechanisms of online grouping and social media identity bubble formation occur across different countries and cultural contexts. One possible explanation for the results may be that, regardless of cultural context, youths encounter gambling-related or otherwise risky content online, making them vulnerable to such risk behaviors [65,66,67]. Alternatively, it is possible that youths who participate in gambling are more likely to seek out similar-minded others online to get validation for their gambling-related identity. Another possible explanation could be that young individuals who engage in gambling activities are more susceptible to becoming involved in social media identity bubbles due to their preferred online interactions. For instance, it is conceivable that young individuals search for this type of risky content and communities built upon gambling activities, which then encourage and support gambling interests and identity [66,69].

When online peer relationships are homophilic and revolve around similar others, it can adversely influence youth behavior, e.g., [30,52]. In terms of this study, we found that when youths were concurrently involved in social media identity bubbles based on shared identity, homophily, and reliance on in-group information, belonging to an online community was associated with higher problem gambling behavior. This supports our second hypothesis. Research has consistently shown that online relationships influence youths’ risk behaviors. More importantly, online relationships influence behavior in contrasting ways than those offline, e.g., [44,45]. The results of this study support these prior findings and suggest that the technologically driven social life of today’s youth and the expanded gambling opportunities are connected. One-sided social interaction without authentic feedback and exposure to contrasting views is biased and may negatively influence youths’ thoughts, beliefs, and decisions concerning their behavior. Consequently, social media identity bubbles may at least partly explain the harmful impact some online relationships have on young individuals. This finding might insinuate that individuals who become involved in social media identity bubbles possess some underlying features motivating them to spend time networking online and prefer interaction with similar-minded others they can identify and agree with. Perhaps these individuals have a greater need to find meaningful connections and identity validation online due to weak social relationships offline.

On the other hand, it should be noted that previous research has suggested that online filter bubbles or echo chambers do not warrant concerns in terms of adverse influences on human behavior [49]. Supporting this notion, prior research has established that online communities can be also beneficial to individuals, providing them support, information, and knowledge [20,22]. Even those online communities that are built upon potentially risky behaviors such as gambling, can have a positive influence on their users. According to a recent systematic review article, gambling-themed online communities can alleviate loneliness among gamblers and help them cope with gambling problems [66]. In the current study, the relationship between identity-driven online interaction and problem gambling was positive but, as indicated by prior literature, the influence of online communities on individuals is manifold.

While research on echo chambers and filter bubbles has sought to explain the impact of personalization of content on political discussion and polarization of opinions, the IBRM is leading the effort to integrate a social psychological perspective into the discussion [27]. By doing so, the IBRM expands our understanding on what the social mechanisms behind online grouping or social media identity bubble forming behavior are, and how individuals perceive their online behavior. By applying the IBRM, this study was able to establish that social media identity bubble involvement may contribute to the existing youth problem gambling phenomenon. It is possible that within social media identity bubbles, risk behaviors such as gambling are viewed as uniting or normal activities. Without alternative feedback and more diverse input, the information and communication young individuals receive about a certain topic remain biased and narrow [30].

Though our study provides additional insight into social media research and the current youth problem gambling discussion, some limitations need to be acknowledged. First, problem gambling was not highly common among our samples, which may be also reflected in our relatively small effect sizes. However, the associations between online belonging, social media identity bubble involvement, and problem gambling were systematically observed across different country samples, indicating that these associations may exist in the general population and outside of clinical samples. Future research should however focus on investigating young problem gamblers specifically to replicate the findings and examine if the results would differ between problem gamblers and youths who are non-problem gamblers or do not gamble at all. Second, our results are based on cross-sectional survey data, which do not allow us to draw possible causal inferences. Future studies utilizing longitudinal methods should investigate whether these associations exist over time.

Our survey did not specifically inquire the participants about their social belonging to a gambling-related online community, but to any online community. This allowed us to investigate the online community’s role in problem gambling at large, but limits our study so that no conclusions can be made about what type of content pertaining to the online communities is related to problem gambling among youth, or if this only applies to those communities that are explicitly related to gambling. Future research should focus on investigating how gambling-themed online communities are related to youth problem gambling. Furthermore, our surveys rely on self-reported information, which is sensitive to social desirability bias, especially when it comes to potentially unfavorable behavior, such as gambling. Lastly, the problem gambling measure SOGS has received some criticism over relatively high rates of false positives in non-clinical samples [104], and its strong focus on items pertaining to borrowing, which insinuates that the scale is inappropriately weighed and not based one theory or empirical data [105]. However, the scale has been used widely in a multitude of research and has shown to be reliable in diverse samples through slight revisions [106] and adjusted cut-off scores [107,108]. Nevertheless, future research should examine the relationships between online belonging, social media identity bubbles, and problem gambling by employing alternative gambling measures.

## 5. Conclusions

This cross-national research used data from four diverse countries; the United States, South Korea, Spain, and Finland to investigate the relationship between two current phenomena: social media identity bubbles and youth problem gambling. Specifically, this study examined if social media identity bubble involvement moderated the relationship between online belonging and problem gambling. Youth problem gambling is an emerging public health and societal issue, and with the changing landscape of advanced technology and human interaction, these two phenomena were expected to be related to one another. We found that strong belonging to an online community was associated with higher problem gambling behavior across three countries, but this was mainly observed among those participants who were concurrently involved in social media identity bubbles. The results of this study provide support to the notion that online relationships influence youths’ risk behaviors. Importantly, our findings further denote that their influence is likely due to social media identity bubbles which are built upon shared identity, homophily, and trust toward information provided by other members of the online clique.

The findings of this study have important implications for preventing youth problem gambling. First, our findings emphasize the increased harms of one-sided online communication. Providing support within an online environment as well as introducing more diverse viewpoints to youth online communication and communities could be effective ways of reaching youths who are susceptible to gambling or currently gamble. Secondly, focusing on building strong offline relationships is recognized as an important element in preventing youth problem gambling.

The identity bubble reinforcement model and the IBR-scale developed to operationalize it fill an empirical and methodological gap concerning social media research. The IBRS is the first measure to emphasize individuals’ own perceptions about their relations to social media networks. This study provides original information on how social media identity bubbles may be influential in online interaction and youth problem gambling behavior.

## Figures and Tables

**Table 1:** Descriptive Statistics.

		United States			South Korea			Spain			Finland	
Variable	M	SD	Range	M	SD	Range	M	SD	Range	M	SD	Range
Problem gambling	1.27	2.55	0–20	0.73	1.92	0–20	1.80	2.91	0–20	1.60	2.56	0–20
Identity bubble *	37.25	13.23	6–60	31.78	11.84	6–60	35.82	11.85	6–60	27.79	9.97	6–60
Online belonging	5.38	2.70	1–10	4.38	2.48	1–10	4.91	2.75	1–10	5.04	2.61	1–10
Offline belonging	20.33	6.70	3–30	20.08	5.86	3–30	21.34	5.81	3–30	20.18	6.13	3–30
Age	20.05	3.19	15–25	20.60	3.24	15–25	20.07	3.16	15–25	21.29	2.85	15–25
Cat. Variables	coding	*n*	%	coding	*n*	%	coding	*n*	%	coding	*n*	%
Gender	male	604	49.83	male	591	49.58	male	621	51.24	male	600	50
	female	608	50.17	female	601	50.42	female	591	48.76	female	600	50

Note. Problem gambling based on the SOGS-scores, measured as a continuous variable. * Social media identity bubble involvement measured with the IBRS–6.

**Table 2:** Correlations of the Main Variables Tested.

		United States			South Korea			Spain			Finland	
Variable	1	2	3	1	2	3	1	2	3	1	2	3
1 Prob. Gambling ^1^	1.00			1.00			1.00			1.00		
2 Identity bubble ^2^	0.0936	1.00		0.1238	1.00		0.1807	1.00		0.0217	1.00	
3 Online belonging	0.1234	0.4847	1.00	0.1480	0.4827	1.00	0.2191	0.4734	1.00	0.0160	0.3583	1.00
4 Offline belonging	−0.0443	0.3491	0.4325	−0.0605	0.2401	0.2316	−0.0555	0.1661	0.2113	−0.0973	0.1811	0.3366

^1^ Problem gambling. ^2^ Social media identity bubble involvement measured with the IBRS–6.

**Table 3:** Linear Regression Models Predicting Problem Gambling from Social Media Identity Bubble Involvement in Four Countries.

	United States			South Korea			Spain			Finland		
**Model 1.**	*b*	SE	*p*	*b*	SE	*p*	*b*	SE	*p*	*b*	SE	*p*
Constant	−1.2	0.59	0.037	1.8	0.50	<0.001	−0.09	0.67	0.894	4.2	0.70	<0.001
Age	0.15	0.02	<0.001	−0.01	0.02	0.431	0.15	0.02	<0.001	0.01	0.03	0.770
Gender	−0.77	0.14	<0.001	−0.58	0.11	<0.001	−1.22	0.16	<0.001	−1.23	0.15	<0.001
Identity bubble *	0.02	0.01	0.012	0.02	0.01	0.003	0.03	0.01	<0.001	0.01	0.01	0.086
Online belonging	0.12	0.03	<0.001	0.10	0.03	<0.001	0.20	0.03	<0.001	0.01	0.03	0.824
Offline belonging	−0.03	0.01	0.014	−0.04	0.01	<0.001	−0.06	0.01	<0.001	−0.07	0.01	<0.001
Adjusted R^2^			0.08			0.06			0.13			0.08
**Model 2.**												
Constant	−0.42	0.64	0.512	−21.6	33.9	0.525	1.2	0.77	0.117	4.8	0.75	<0.001
Age	0.15	0.02	<0.001	0.01	0.02	0.485	0.14	0.02	<0.001	0.01	0.03	0.703
Gender	−0.77	0.14	<0.001	−0.59	0.11	<0.001	−1.2	0.16	<0.001	−1.2	0.15	<0.001
Identity bubble *	−0.04	0.05	0.464	−0.03	0.05	0.603	−0.04	0.07	0.588	−0.01	0.02	0.452
Online belonging	−0.02	0.06	0.761	−0.07	0.06	0.251	−0.08	0.09	0.355	−0.12	0.08	0.111
Identity bubble X online belonging	0.02	0.01	0.010	0.03	0.01	0.003	0.04	0.01	0.001	0.00	0.00	0.063
Offline belonging	−0.03	0.01	0.012	−0.04	0.01	<0.001	−0.06	0.01	<0.001	−0.07	0.01	<0.001
Adjusted R^2^			0.08			0.06			0.14			0.08

Note. Gender reference group male. * Social media identity bubble involvement measured with the IBRS–6. Significance level at *p* < 0.05.
